# Hypothermia reduces glymphatic transportation in traumatic edematous brain assessed by intrathecal dynamic contrast-enhanced MRI

**DOI:** 10.3389/fneur.2022.957055

**Published:** 2022-10-20

**Authors:** Yingnan Bai, Mingyuan Yuan, Honglan Mi, Fengchen Zhang, Xiangyu Liu, Chen Lu, Yinghui Bao, Yuehua Li, Qing Lu

**Affiliations:** ^1^Department of Cardiology, Shanghai Institute of Cardiovascular Diseases, Zhongshan Hospital and Institute of Biomedical Sciences, Fudan University, Shanghai, China; ^2^Department of Radiology, Affiliated Zhoupu Hospital, Shanghai University of Medicine and Health Sciences, Shanghai, China; ^3^Department of Radiology, Charité-Universitätsmedizin Berlin, Berlin, Germany; ^4^Department of Neurosurgery, Ren Ji Hospital, Shanghai Jiao Tong University School of Medicine, Shanghai, China; ^5^Department of Radiology, Ren Ji Hospital, Shanghai Jiao Tong University School of Medicine, Shanghai, China; ^6^Shanghai Wei Yu International School, Shanghai, China; ^7^Department of Radiology, Affiliated Sixth People's Hospital, Shanghai Jiao Tong University, Shanghai, China; ^8^Department of Radiology, Shanghai East Hospital Tongji University, Shanghai, China

**Keywords:** hypothermia, glymphatic function, traumatic brain injury, brain edema, DCE-MRI, diffusion-weighted imaging (DWI)

## Abstract

The glymphatic system has recently been shown to clear brain extracellular solutes and can be extensively impaired after traumatic brain injury (TBI). Despite hypothermia being identified as a protective method for the injured brain via minimizing the formation of edema in the animal study, little is known about how hypothermia affects the glymphatic system following TBI. We use dynamic contrast-enhanced MRI (DCE-MRI) following cisterna magna infusion with a low molecular weight contrast agent to track glymphatic transport in male Sprague–Dawley rats following TBI with hypothermia treatment and use diffusion-weighted imaging (DWI) sequence to identify edema after TBI, and further distinguish between vasogenic and cytotoxic edema. We found that hypothermia could attenuate brain edema, as demonstrated by smaller injured lesions and less vasogenic edema in most brain subregions. However, in contrast to reducing cerebral edema, hypothermia exacerbated the reduction of efficiency of glymphatic transportation after TBI. This deterioration of glymphatic drainage was present brain-wide and showed hemispherical asymmetry and regional heterogeneity across the brain, associated with vasogenic edema. Moreover, our data show that glymphatic transport reduction and vasogenic edema are closely related to reducing perivascular aquaporin-4 (AQP_4_) expression. The suppression of glymphatic transportation might eliminate the benefits of brain edema reduction induced by hypothermia and provide an alternative pathophysiological factor indicating injury to the brain after TBI. Thus, this study poses a novel emphasis on the potential role of hypothermia in managing severe TBI.

## Introduction

Traumatic brain injury (TBI) is a life-threatening condition in which a detrimental complication develops secondary to cerebral edema. The high water content in the posttraumatic brain expands the brain volume, leads to increased intracranial pressure, impairs cerebral perfusion and oxygenation, and contributes to additional ischemic injuries (1). Suppressing edema represents a promising therapeutic strategy as the severity of swelling predicts long-term functional outcomes.

Hypothermia has been identified as a protective method for the injured brain because it affects many pathophysiological processes, including decreasing edema formation (2, 3). However, definite effects of hypothermia on outcomes in TBI were not confirmed in multiple randomized controlled clinical trials (4–8). The role of hypothermia in treating TBI should be further investigated.

Classically, there are two major types of traumatic brain edema: vasogenic edema, due to blood-brain barrier (BBB) disruption resulting in extracellular water accumulation, and cytotoxic edema, due to sustained intracellular water collection (9). Recent evidence suggest that edema formation following TBI is also associated with perivascular spaces (10–12), which have recently been described as a system to clear interstitial waste from the brain interstitium and is called the “glymphatic system” (13, 14). CSF could be shifted from the cerebral cisterns through the perivascular spaces to the brain leading to severe brain swelling and allowing the intra-cerebral pressure to rise steeply following TBI (10, 14, 15). However, little is known about whether and how hypothermia affects the glymphatic system and improves CSF-ISF (interstitial fluid) exchange to eliminate brain edema formation in the early stage of TBI.

The diffusion changes of water in cytotoxic or vasogenic edema induced by TBI and minimized by hypothermia could be detected by magnetic resonance diffusion-weighted imaging (DWI) (9, 16). Non-invasive DWI quantifies the diffusion of water in the brain and provides an understanding of trauma-related cerebral edema formation (17). The glymphatic transportation following neurodegenerative disorders can be measured using intrathecal dynamic contrast-enhanced MRI (DCE-MRI) (13, 18, 19), a well-established and clinically relevant imaging technique. We use DCE-MRI following cisterna magna infusion with a small molecular weight contrast agent to track glymphatic transport in TBI with hypothermia treatment. Together with DWI, we will investigate (1) the role of the glymphatic system in the formation of TBI-related brain edema and (2) whether and how hypothermia can impact brain edema via the glymphatic system.

## Materials and methods

### Animals and groups

**Figure 1:**
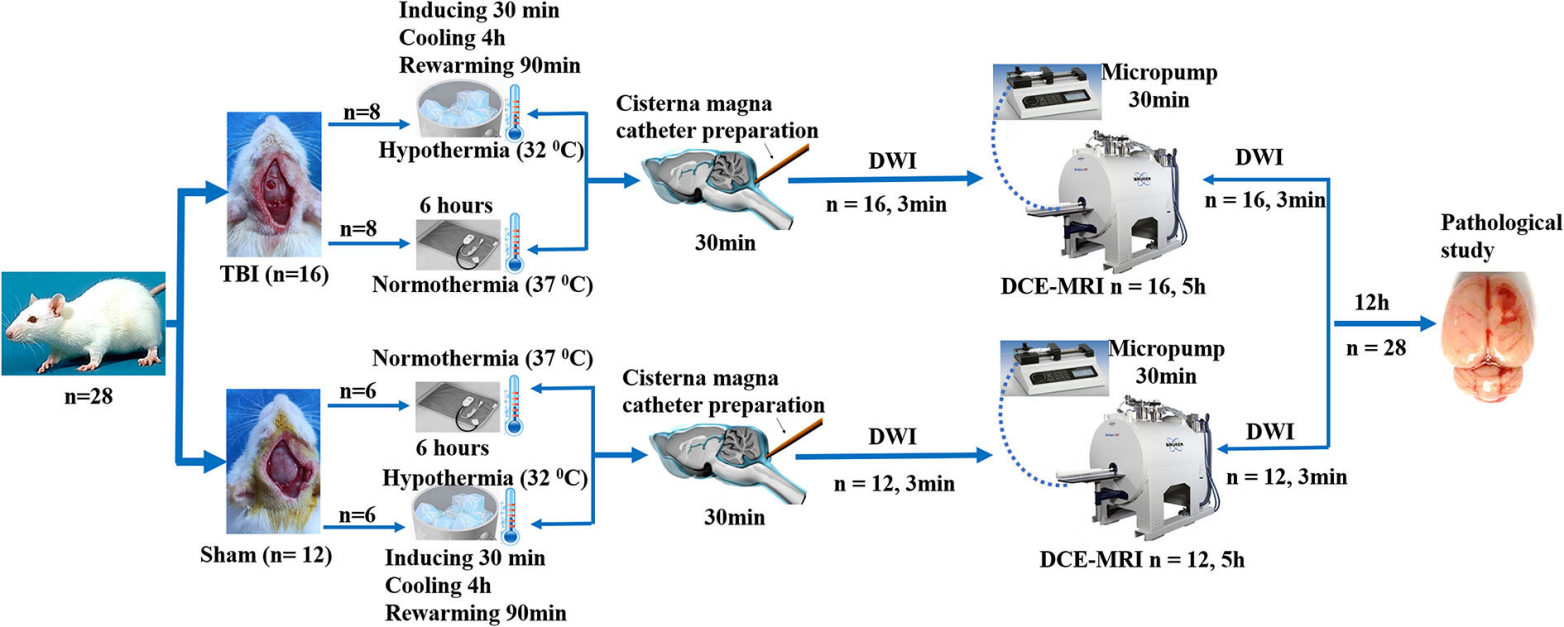
Timeline schematic of the experimental layout where animals received TBI and Sham surgery, treated with hypothermia and normothermia, and then performed with diffusion-weighted imaging (DWI) sequences and a dynamic contrast-enhanced MRI (DCE-MRI) following intra-cisterna Magna injection of low molecular weight paramagnetic contrast agent.

Male Sprague–Dawley rats (280–320 g), 3 months of age (*n* = 28), were randomly divided into four groups: sham injury with normothermia (Sham-NT, 37°C; *n* = 6); sham injury with hypothermia (Sham-HT, 32°C; *n* = 6); TBI with normothermia (TBI-NT, 37°C; *n* = 8); and TBI with hypothermia (TBI-HT, 32°C; *n* = 8). After the temperature management, each group underwent magnetic resonance (MR) examination. Twelve h after imaging, all surviving animals were sacrificed, and their brains were removed for immunostaining analyses. An experimental procedure and timeline schematic are described in Figure 1. Rats were placed in individual cages and accessed freely to food and water in an animal facility where temperature and humidity were controlled. All animal procedures were approved by the animal care and experimental committee of the School of Medicine of Shanghai Jiao Tong University.

### Brain injury surgical preparation and severe CCI TBI

Surgical preparation and cortical controlled impact model (CCI) for TBI were performed as previously described with slight modification (20). In brief, the rats were mounted in a stereotaxic frame after initially anesthetized with 4% isoflurane and maintained with 1.5–2.0% isoflurane. A midline incision (2 cm) was made to expose the skull over the cranial vault after shaving and disinfecting the skin. A craniectomy of 4 mm was performed on the right parietal bone (midway between bregma and lambda), and the bone flap was gently removed to keep the underlying dura intact. Animal CCI injury was performed using an electromagnetically controlled impacting device (Pin-Point™ PCI3000 Precision Cortical Impactor™, Hatteras Instruments, Cary, USA). A severe cortical contusion injury was induced with a strike velocity of 3.0 m/s, a deformation depth of 2.0 mm, and a dwell time of 180 ms using a 3.0 mm rounded impacting tip that angled vertically toward the exposed brain dura. Only the animals in the TBI group were subjected to CCI injury. The rat received an identical procedure except for CCI injury in the sham injury group. Sixteen rats subjected to CCI and 12 to the sham operation were randomly assigned to the normothermia or hypothermia treatment.

### Hypothermia treatment

Hyperthermia was induced by immersing the body of the anesthetized rat in ice-cold water. A plastic bag was used to protect the animal's skin and fur from the water before immersion (head exposed). A target brain temperature of 32°C was achieved for ~30 min and was maintained for 4 h under general anesthesia at room temperature by intermittent application of ice packs as needed. Gradual rewarming to normothermia levels (37°C) was done for 90 min to avoid rapid rewarming. Using a heating blanket, the normothermic treatment group achieved a brain temperature of 37°C. To manipulate the temperature adjustment, the brain temperature was monitored with a digital electronic thermometer (model DP 80; Omega Engineering, Connecticut). A temperature probe (model HYP-033-1-T-G-60-SMP-M; Omega Engineering, Connecticut) was inserted 5.0 mm ventrally into the surface of the skull. The probe was replaced immediately after the injury. Rectal temperatures and other physiologic variables were also monitored throughout the procedure.

### Cisterna magna catheter preparation

Following the complete normothermic or hypothermic procedure (~6 h after TBI or sham injury operation), contrast-infusion catheter placement was performed after rats were anesthetized with 3% isoflurane. The animal was positioned in a stereotaxic frame with the head flexed to 50°. A small incision was made to expose the dura mater following the atlantooccipital membrane exposure. A PE-10 catheter filled with normal saline was advanced 1 mm into the intrathecal space through the dura mater incision. The incision was sealed with cyanoacrylate glue around the catheter. The intrathecal catheter was connected to the long PE-10 line filed with paramagnetic MR contrast diluted in 0.9% NaCl when rats were transferred to the MRI instrument. A microinfusion pump with a 1-cc syringe (Baxter Model AS50 Infusion Pump, Baxter Healthcare Corporation, Shanghai, China) was attached to the PE-10 line. During image acquisition, anesthesia was maintained with a 1.5–2.0% isoflurane mixture delivered by a nose cone. The animal body temperature was monitored and kept within a range of 36.5–37.5°C using a feedback-controlled water bath. The respiratory and heart rate were continuously monitored using an MRI-compatible system (SA Instruments, Stony Brook, NY, USA).

### MRI acquisitions

All MRI acquisitions were performed on a Bruker 7.0T/20 magnet (Bruker BioSpin, Billerica, MA). All rats were imaged supine and fixed with a stereotaxic ear bar to immobilize the head. To acquire the whole-brain image with high quality, we used a 3-cm planar receive surface radio-frequency coil (Bruker) as a receiver and a birdcage-type coil as a transmit RF coil.

All rats underwent the identical whole-brain MRI protocol immediately after temperature management. A DCE-MRI sequence, 3D T1-weighted imaging (T1WI), was acquired in the axial plane before and after contrast agent infusion to visualize the glymphatic transport. A low molecular weight paramagnetic contrast agent (30 mM/50 μl of Gd-DTPA, Magnevist, MW 938Da; Bayer HealthCare Pharmaceuticals Inc.) was delivered intrathecally via the indwelling catheter at a constant infusion rate of 1.6 μl/min over 30 min (13, 21). The 3D T1WI sequence was repeatedly acquired 5 h with following parameters: TR = 18 ms, TE = 4 ms, flip angle =12°, FOV = 32 × 16 × 16 mm^3^, matrix = 256 × 128 × 128. For detection of brain tissue changes after temperature management, a whole-brain DWI study was performed before and immediately after the DCE-MRI study, ~12 h post-TBI, with the following parameters: TR=4,000 ms, TE =36 ms, FOV =32 × 32 mm^2^, matrix=64 × 64, 15 slices, thickness =1 mm, interslice distance 0.0 mm, b value=50, and 1,000 s/m^2^. T2-weighted imaging (T2WI) (TR=4,000 ms, TE = 30 ms, FOV =32 × 32 mm^2^, matrix= 128 × 128, 15 slices, thickness = 1 mm) were also performed before the DCE-MRI study.

## MRI post-processing and analysis

### DWI analysis for traumatic lesion volume and edema quantification

**Figure 2:**
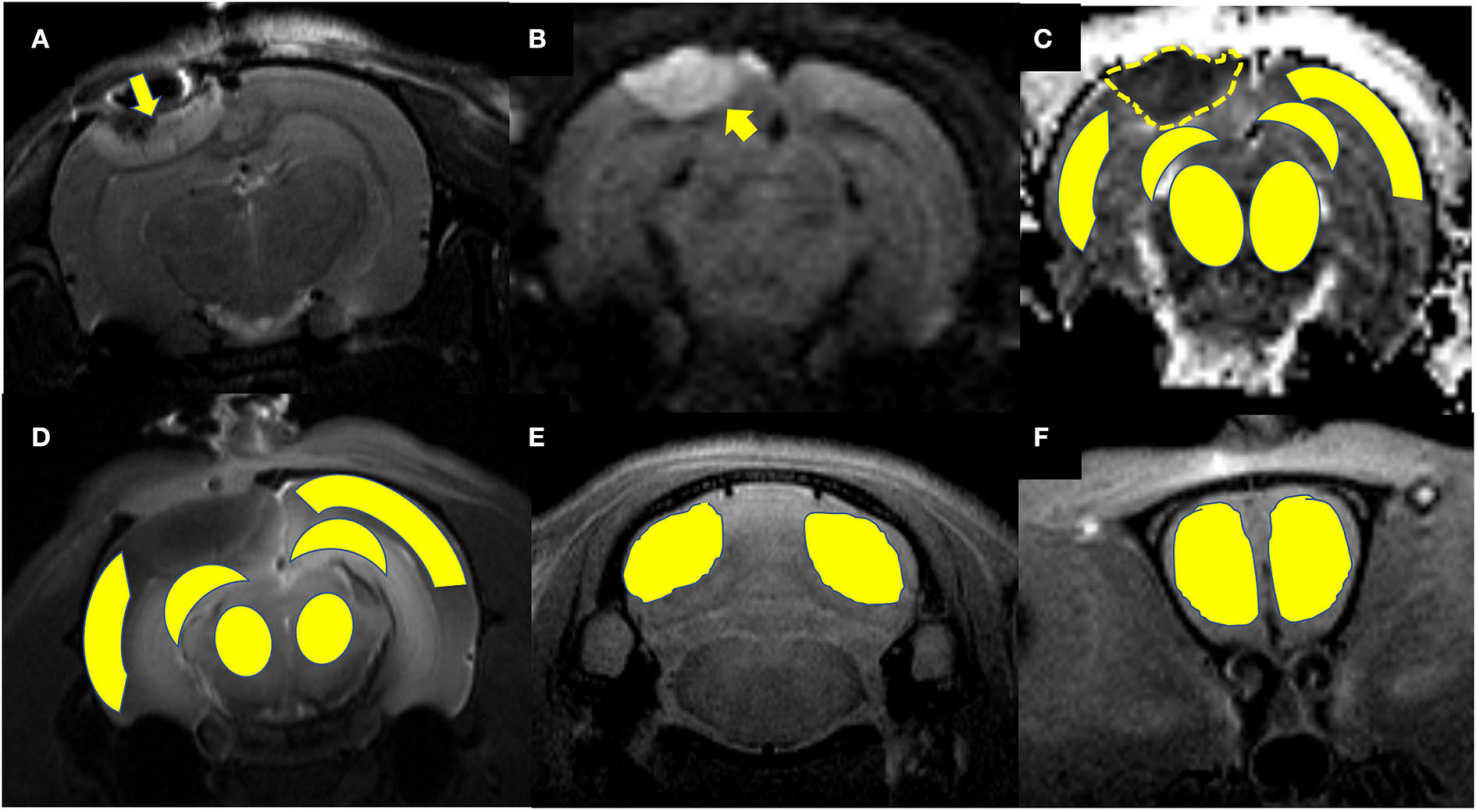
Representative images describing the creation of regions of interest (ROIs) for obtaining signal intensity (SI) changes before and after intrathecal contrast agent delivery and for obtaining the diffusivity of the brain subregions before and after temperature management. **(A)** Coronal T2WI and **(B)** axial DWI are used as a reference to direct the ROIs creation in the brain subregion on T1WIs and ADC maps. A traumatic lesion (yellow arrow) and bilateral cortex, hippocampus, and thalamus can be visualized. **(C)** Depicts the diffusivity of traumatic lesions and subregions obtained from the ADC map. The yellow dashed line defines the area of edema as marked manually by an independent observer. The area measurement on a series of slices was followed by calculating edema volume by defined slice thickness. **(D)** The signal intensity change after Magnevist delivery is obtained from the brain subregion, including bilateral cortex (ROI in injured site excludes the lesion), hippocampus, thalamus, and **(E)** cerebellum, and **(F)** olfactory bulb on coronal T1WI sections.

Maps of the mean diffusivity (ADC-map) were derived using the standard Paravision 6.0.1 software (Bruker) algorithm. Edematous tissue of traumatic lesions appears hyperintense in diffusion-weighted MRI and shows hypointense in ADC maps (Figures 2B,C). To quantify the diffusivity of edematous tissue, edema was defined as the ADC value in a pixel dropping or rising three standard deviations from baseline. In each brain image of TBI before temperature management, a circular ROI was placed in the uninjured cortex on the contralateral side to serve as a baseline tissue ROI. Matching ROI was manually created with the Paravision 6.0.1 ROI tool (example presented in Figure 2C). The lesion's resulting size measurements (mm^2^) were exported, and the total volume of the injured lesions was calculated from the areas on the single maps and the thickness of the scan slices. For diffusivity analysis in the brain subregion, ROIs encompassing the ipsilateral and contralateral brain subregion, including cortex (excluding injured lesion), hippocampi, thalamus, and olfactory bulb cerebellum, were bilaterally created on the fixed axial sections of ADC maps (Figure 2C). The mean diffusivity obtained from three sequential anatomic ADC maps was recorded as the corresponding subregion diffusivity. The ADC map of the Sham group was acquired as a reference.

### Gd-DTPA MRI data processing and analysis

MRI postprocessing and data analysis were performed in a blinded fashion using an open-source software 3D slicer (https://www.slicer.org/, version 4.10) by a radiologist and a neurologist. Both of them had experience in animal TBI studies. They resolved disagreement by consensus. The detailed MRI data processing and parameter calculation methods have been previously described (13, 21, 22). In brief, the time series 3D T1WI brain data were strictly registered to a standard reference template for head motion correction. The conversion of the voxel-by-voxel signal intensity (SI) change on contrast-enhanced images was achieved by subtracting and dividing contrast-enhanced images at each time point by the baseline images. A simulated time-intensity curve (TIC) was obtained from the signal intensity change in every region of interest (ROI) manually created on the baseline and contrast-enhanced MRI images over time. To quantify the glymphatic transport of contrast agents in the brain subregions, ROIs were bilaterally created on the axial 3D T1WIs (Figures 2D,F) covering each brain subregion, including the cortex and hippocampi thalamus, olfactory bulb, and cerebellum. The area of traumatic lesion was excluded from cortical ROIs referring to T2WI (Figures 2A,B) when cortical ROIs were drawn in the injured hemisphere. The semi-quantitative kinetic parameters characterizing the contrast infusion and cleanout in brain tissue, including infusion rate, clearance rate, and clearance time constant, were calculated from the average TICs. These parameters were computed from the following equations:


$$\begin{array}{l} Infusionrate=\left[\left(SIpeak-SIpre\right)/\left(SIpre\times Tpeak\right)\right]\times 100\;\left(\%\right) \end{array}$$

$$\begin{array}{l} Clearancerate=[((SIpeak-SIend)/(SIpre\times Tend)]\times 100(\%) \end{array}$$

$$\begin{array}{l} Clearancetimeconstant=AUC/Clearancerate \end{array}$$

SIpeak: signal intensity of peak enhancement, SI pre: signal intensity before injection, SIend: signal intensity of Tend, Tpeak: time to peak enhancement, Tend: the time at the end of acquisition, AUC, area under the TIC in the relaxing phase.

### Immunohistochemistry and image analysis

The animal brains were quickly removed after anesthetized and perfused with 500 mL of 4% paraformaldehyde. The brains were fixed in 4% paraformaldehyde at 4°C for 20 h, then immersed in 30% sucrose in 0.1 M PBS at 4°C overnight and subsequently embedded in paraffin. Coronal sections with 4 μm thickness were deparaffinized and rehydrated in gradient alcohol to examine histopathological endpoints in the brain. Sections from three coronal levels of the brain were examined at ~3.0 mm posterior to bregma (area underlying the injury site), middle of the cerebellum, and olfactory bulb. Slides were then placed in the microwaveable vessel filled with the sodium citrate buffer (10 mM Sodium Citrate, 0.05% Tween 20, pH 6.0) for heat-induced epitope retrieval by a microwave. The sections were then blocked and permeabilized for 1.5 h (1% BSA, 1% normal goat serum, and 0.3% Triton) and incubated for 24 h at 4°C with primary antibodies, rabbit anti-aquaporin-4(AQP_4_) (AF5164, 1:200; Affinity Biosciences, USA). After rinsing sections three times for 5 min each, sections were incubated with HRP-conjugated secondary antibody (S0001, 1:200; Affinity Biosciences, USA) at 37°C for 40 min and proceeded with chromogen by using DAB Kit. Hematoxylin was applied as a counterstain. Sections were dehydrated through successive ethanol solutions, cleared in xylene, and coverslipped using xylene-based mounting media. Image capture and analysis were achieved using a 3DHISTICH Pannoramic 250 Digital Slide Scanner (20X, Thermo Fisher Scientific) and HALO image analysis software (version 2.4.0.119028, Indica Labs). Immunoactivity quantification for each animal in brain subregions including cortex, hippocampus, thalamus, cerebellum, and olfactory bulb was determined using the mean area of positive staining (23).

### Statistical analysis

All values are expressed as mean ± SD. Data were statistically evaluated and plotted in the Prism software (Graphpad, La Jolla, CA, USA). Comparisons between groups were made by one-way analysis of variance (ANOVA) with Bonferroni's *post-hoc* test for between-group comparison. Mann–Whitney *U*-test was used for two groups compared with variance heterogeneity. The correlation between AQP_4_ expression level, glymphatic drainage function, and ADC value were performed with Spearman correlation analysis. For all statistical tests, a *P*-value < 0.05 was considered significant.

## Results

All rats included in the study remained physiologically stable during the temperature modulation and imaging experiment, with a normal range of heart and respiratory rates and body temperature of 36.5–37.5°C. Several major representative anatomical structures, such as the cortex, hippocampus, thalamus, olfactory bulb, and cerebellum, could be identified on DWI or 3D T1WI (Figure 2).

### Effect of hypothermia on brain edema in TBI rat

**Figure 3:**
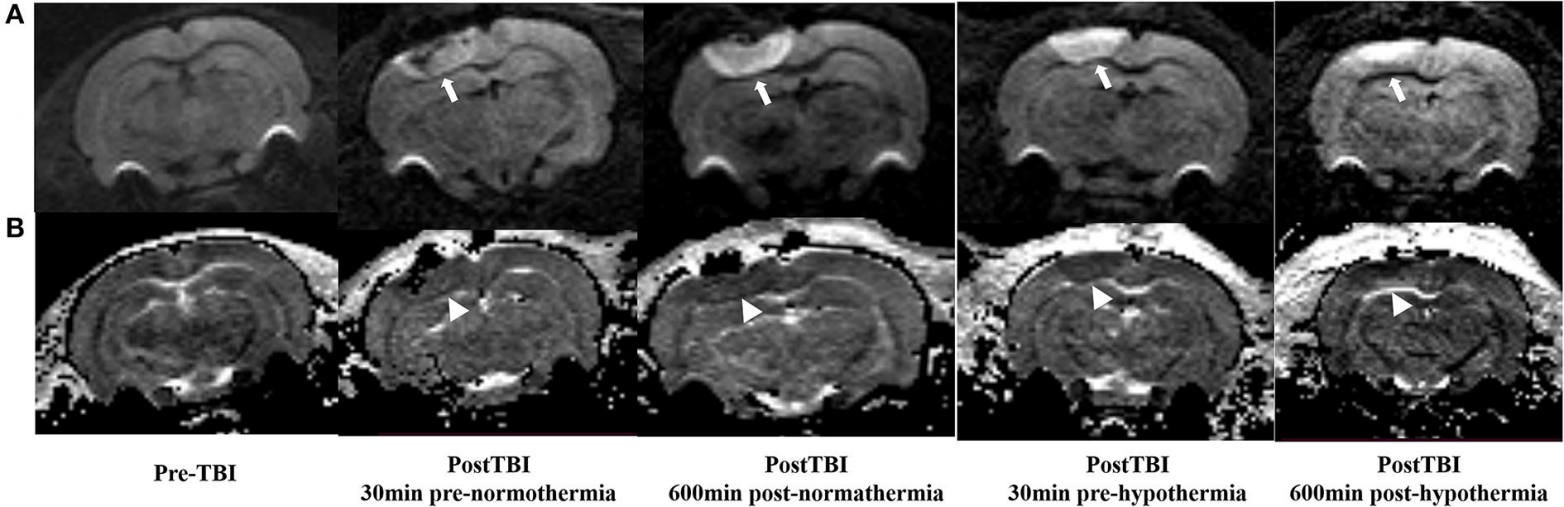
DWI and calculated ADC maps acquired before and 30 min, 10 h post-TBI. Edematous tissue of traumatic lesion appears hyperintense in diffusion-weighted MRI [**(A)**, arrows] and shows hypointense in ADC maps [**(B)**, arrowheads].

**Figure 4:**
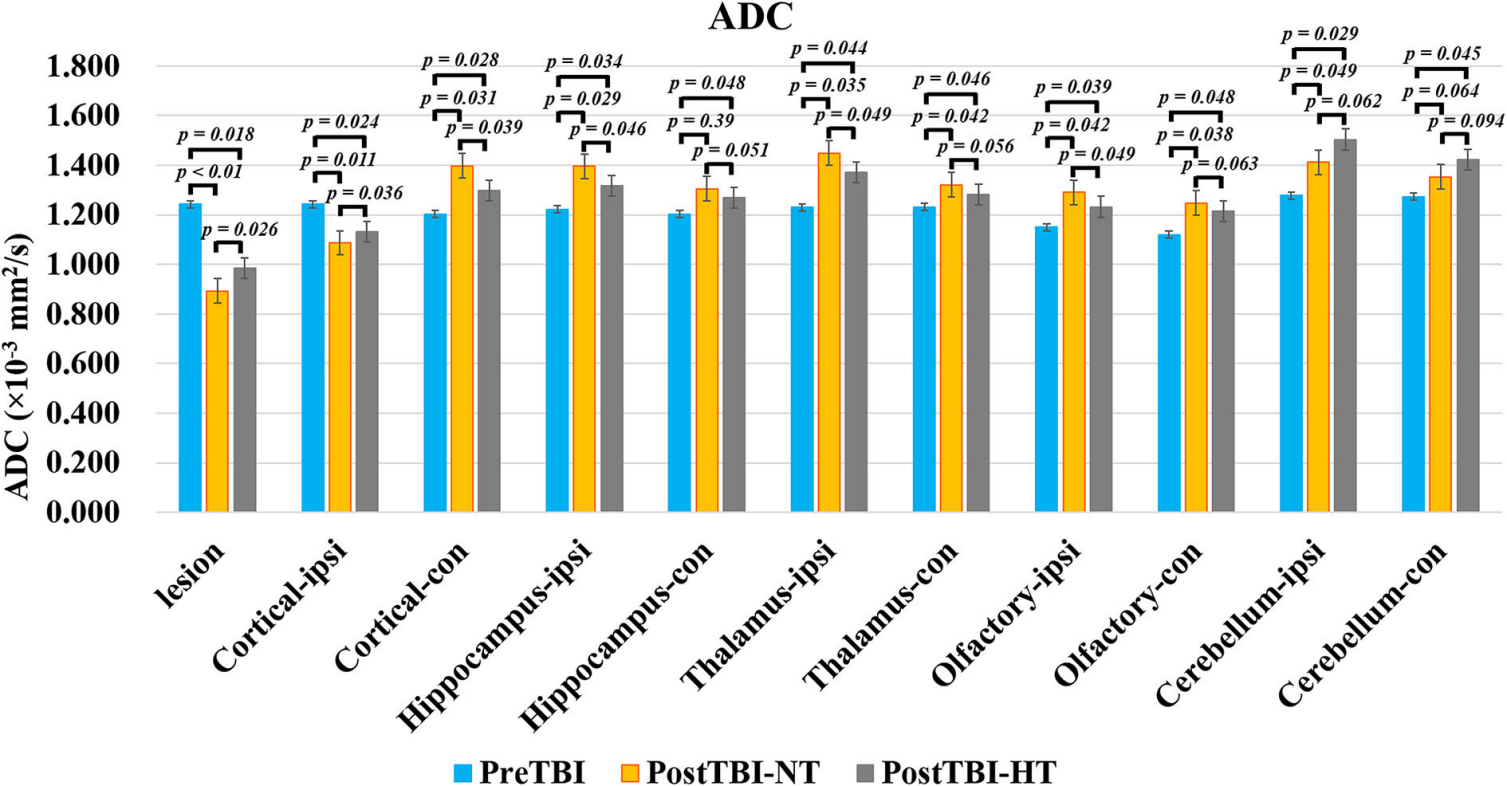
The quantitative analysis of ADC value on ipsilateral and contralateral brain subregion of TBI impacted by hypothermia and normothermia. The ADC map was derived from the DWI sequence acquired before TBI and 12 h after. Data in **Figure 11**: TBI-NT *n* = 8, TBI-HT *n* = 8. All n values refer to the number of rats used, and the error bars depict the mean ± s.e.m. One-way ANOVA calculated *P*-values with Bonferroni's multiple comparison test, and *P* < 0.05 indicates significance. Contra, contralateral hemisphere; ipsi, ipsilateral hemisphere.

**Figure 5:**
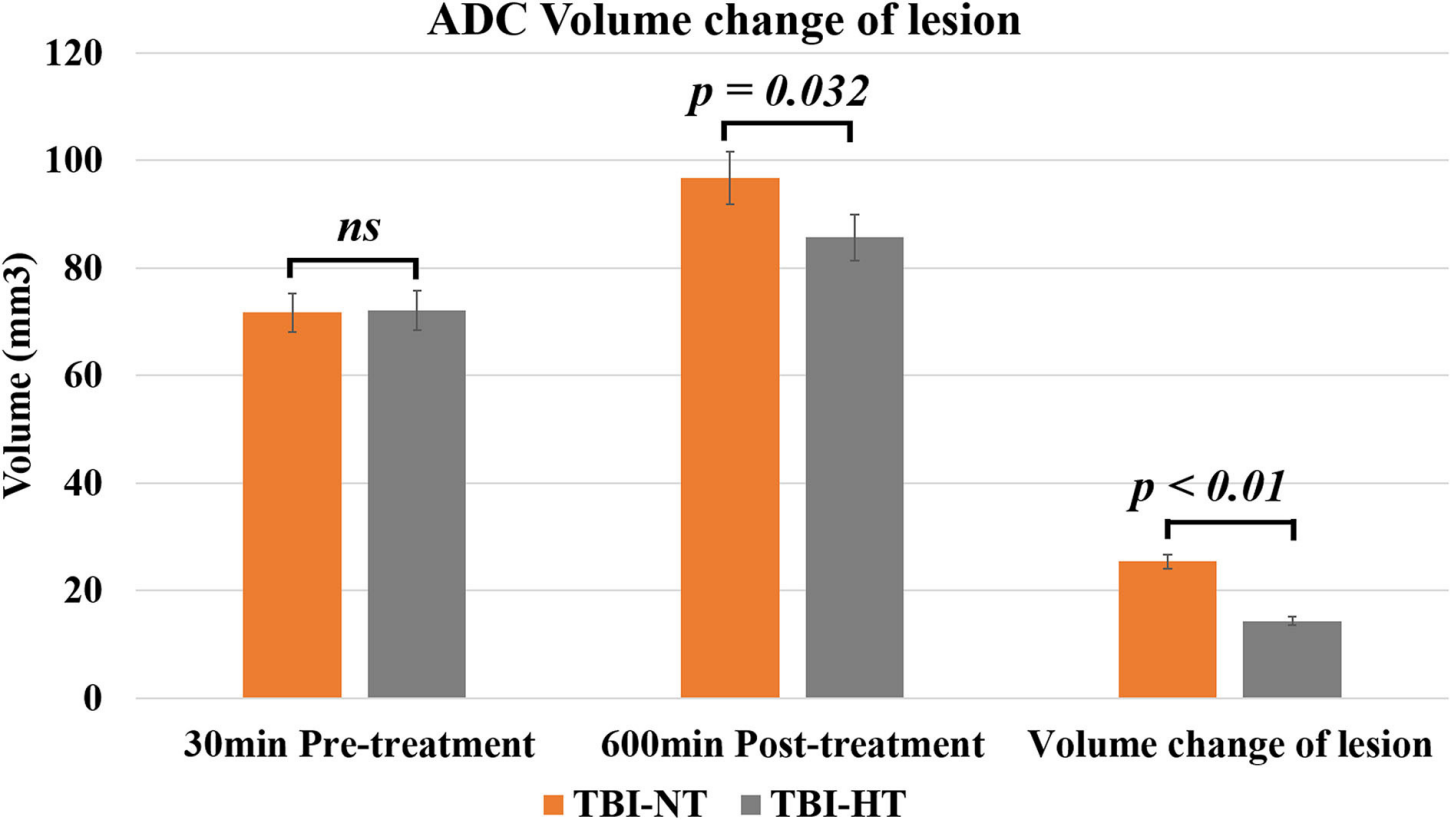
The histogram depicts the ADC volume difference of traumatic lesions before and 10 h after therapeutic hypothermia or normothermia. Data in Figure 5: TBI-NT *n* = 8, TBI-HT *n* = 8. All *n* values refer to the number of rats used, and the error bars depict the mean ± s.e.m. *P*-values were calculated by the Mann–Whitney *U-*test, and *P* < 0.05 indicates significance.

The ADC results obtained from DWI before and after hypothermic or normothermic management in four groups were presented in Tables 1, 2 and Figures 3–5. Immediately after TBI and 10 h later, the traumatic lesion in the cortex showed hyperintensity on DWI (Figure 2B) and hypointensity on ADC in two TBI groups (Figure 3). The ADC value of the lesions and ipsilateral adjacent cortex was significantly lower than that of the corresponding cortex area before CCI, which is found in both TBI-HT and TBI-NT (Table 1, Figure 4). Compared to the TBI-NT group, the diffusivity changes in the injured cortex and adjacent cortex in the TBI-HT group were limited, demonstrated by less decreased ADC value (Table 1, Figure 4) and minor lesion volume change in 10 h (Table 2, Figure 5). However, the ADC value showed an increase in other brain subregions, including the contralateral cortex, bilateral hippocampus, thalamus, cerebellum, and olfactory bulb in TBI groups, compared to the control groups (Table 1 and Figure 4). The diffusivity change in these brain subregions was also affected by hypothermia, demonstrated by less increased ADC value (Table 1, Figure 4). Moreover, the diffusivity change induced by TBI and hypothermia showed a hemisphere difference. The ADC values in contralateral subregions were lower than in ipsilateral subregions, although some did not show a significant difference (Table 1, Figure 4).

**Table 1:** ADC value changes before TBI and 10 h after TBI in rats treated with therapeutic hypothermia or normothermia.

**Brain regions/groups**	**lesion**	**Cortical**		**Hippocampus**		**Thalamus**		**Olfactory bulb**		**Cerebellum**	
	**TBI**	**IPSI**	**Contra**	**IPSI**	**Contra**	**IPSI**	**Contra**	**IPSI**	**Contra**	**IPSI**	**Contra**
PreTBI	1.242 ± 0.090	1.243 ± 0.094	1.203 ± 0.056	1.223 ± 0.098	1.203 ± 0.109	1.229 ± 0.171	1.232 ± 0.150	1.12 ± 0.134	1.15 ± 0.111	1.277 ± 0.107	1.273 ± 0.162
TBI-NT	0.892 ± 0.253	1.088 ± 0.150	1.398 ± 0.154	1.396 ± 0.246	1.305 ± 0.154	1.448 ± 0.197	1.321 ± 0.142	1.291 ± 0.092	1.247 ± 0.134	1.412 ± 0.179	1.352 ± 0.147
TBI-HT	0.984 ± 0.187	1.132 ± 0.229	1.297 ± 0.148	1.317 ± 0.190	1.268 ± 0.189	1.370 ± 0.280	1.281 ± 0.203	1.232 ± 0.167	1.215 ± 0.156	1.503 ± 0.315	1.422 ± 0.270

**Table 2:** ADC volume changes of traumatic lesion before and 10 h after therapeutic hypothermia or normothermia.

**Volume (mm^3^)**	**30 min pre-treatment**	**600 min post-treatment**	**Volume change of the lesion**
TBI-NT	71.67 ± 11.35	96.72 ± 20.35	25.42 ± 6.37
TBI-HT	72.13 ± 10.91	85.67 ± 13.21	14.38 ± 4.22

### Effect of hypothermia on brain-wide Gd-DTPA transport in TBI rat

**Figure 6:**
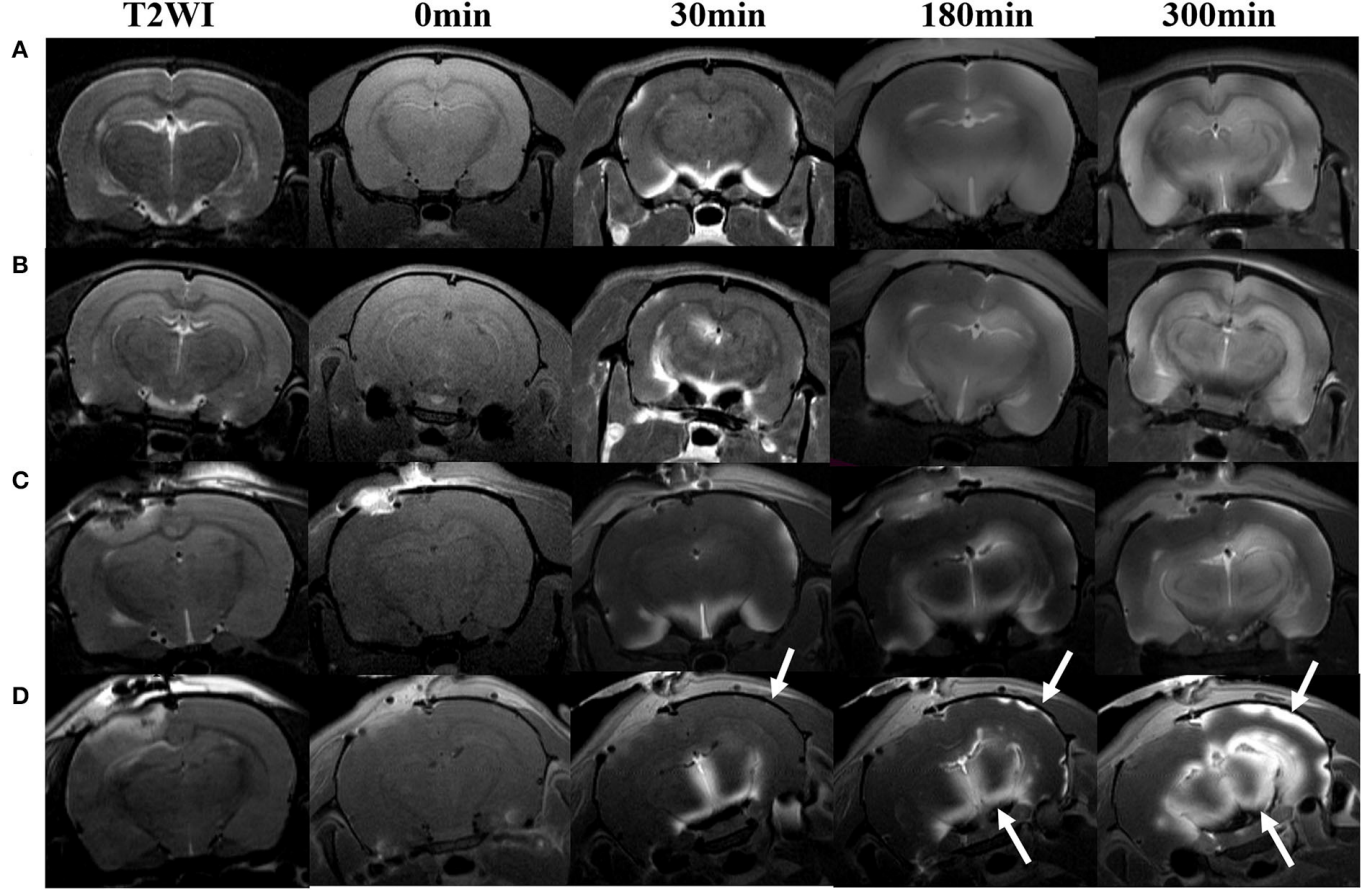
Hypothermia-induced dynamic contrast concentration changes in traumatic and sham injury brain after intrathecal infusion of low molecular weight contrast agent Magnevist. The time series of coronal T1-weighted MRI images demonstrates early influx (0–30 min), deep anatomic enhancement 180 min, and 5 h in subregion including bilateral cortex, bilateral hippocampus, and bilateral thalamus in Sham-NT **(A)**, Sham-HT **(B)**, TBI-NT **(C)**, and TBI-HT **(D)** animal brains. Coronal T2WI is used as a reference to visualize brain subregions, such as the cortex, hippocampus, and thalamus on T1WIs. As indicated by arrows, therapeutic hypothermia leads to limited contrast agent diffused into the cortex at 30 min, restricted to the superficial cortex and partial thalamus at 180 min, and incompletely whole-brain infused at the end of the experiment (300 min) after Gd-DTPA infusion in the brain of TBI-HT rat. Asymmetrical hemisphere distribution of contrast is detected in the injured brain of TBI-NT **(C)** and TBI-HT **(D)** rats.

**Figure 7:**
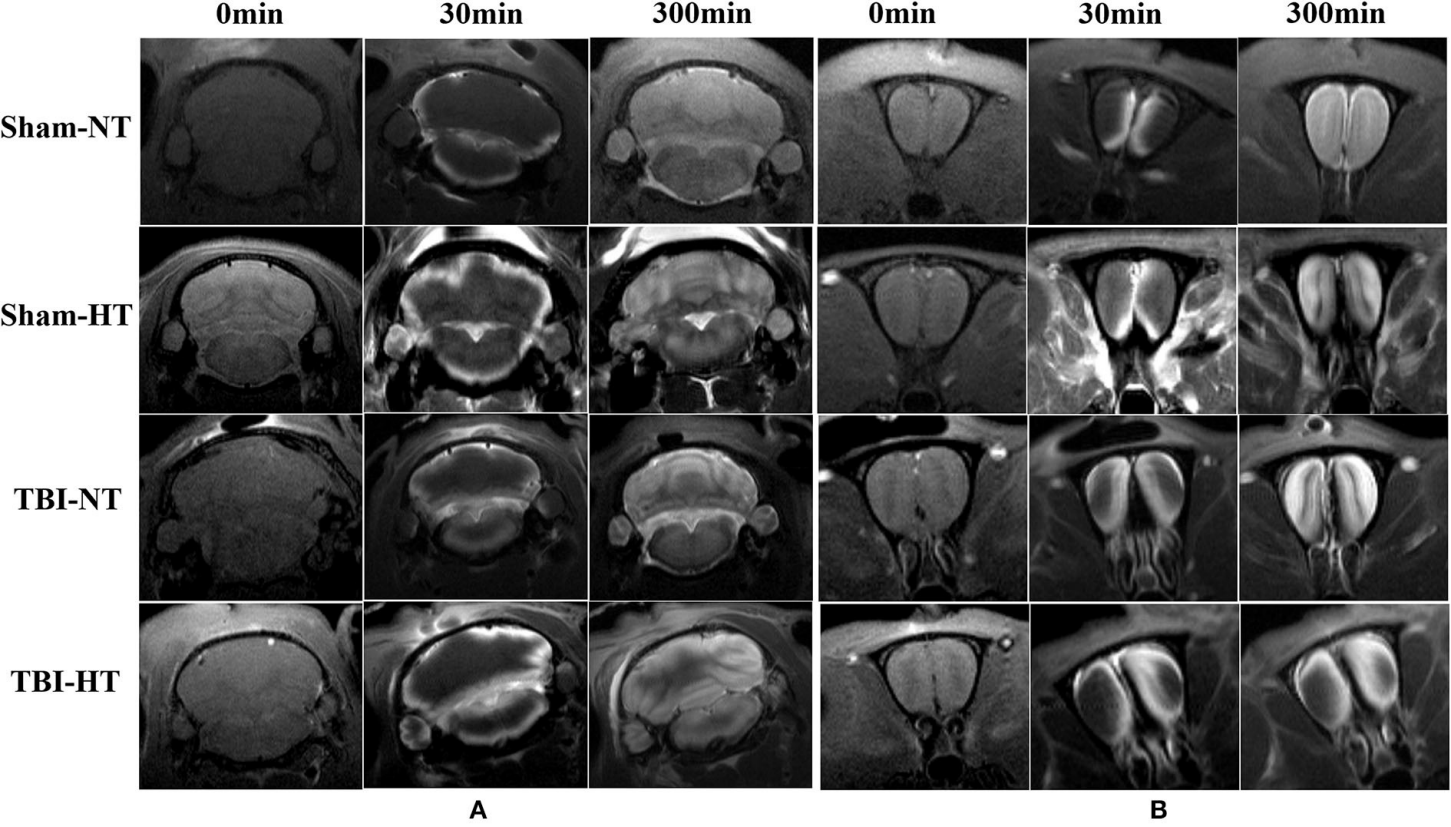
Contrast distribution in the cerebellum and olfactory bulb of TBI and sham injury rats after intrathecal infusion of low molecular weight contrast agent Magnevist. **(A)** The time series of coronal T1-weighted MRI images demonstrates a symmetrical distribution pattern with different infusion speeds in cerebellum of Sham-NT, Sham-HT, TBI-NT, and TBI-HT animal brain from early influx (0–30 min) to the end of the experiment (300 min). **(B)** Besides different infusion speeds, an asymmetrical distribution pattern is also detected in the olfactory bulb in TBI-NT and TBI-HT, but not in sham injury controls during the same experiment period.

**Figure 8:**
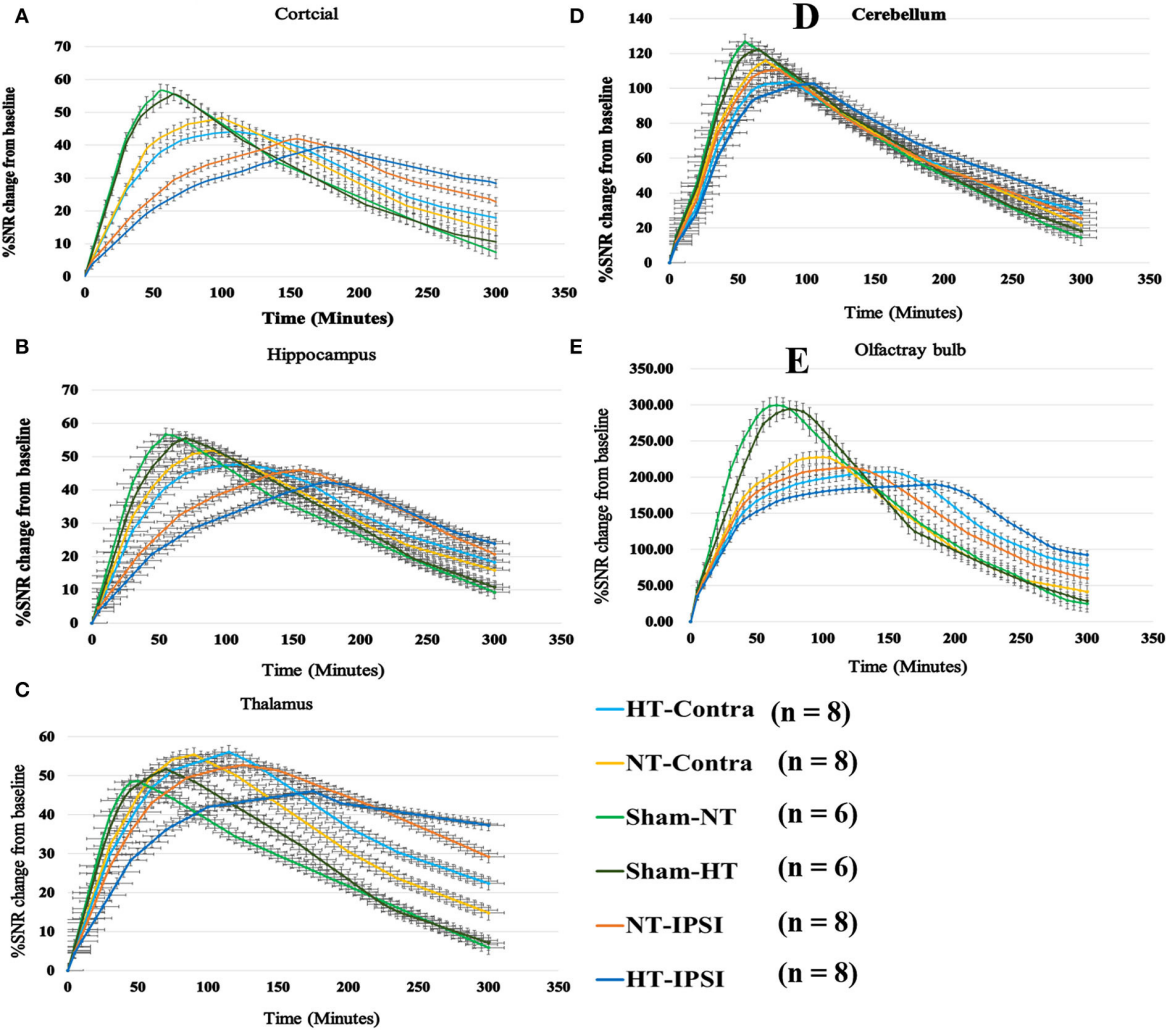
Group average TICs derived from dynamic time series of T1WI with Gd-DTPA. The SNR% changes from baseline along time obtained from coronal T1WI sections encompassing bilateral brain subregion including ipsilateral and contralateral cortex **(A)**, ipsilateral and contralateral hippocampus **(B)**, ipsilateral and contralateral thalamus **(C)**, ipsilateral and contralateral cerebellum **(D)**, and ipsilateral and contralateral olfactory bulb **(E)**. Within the major experimental period, longer to peak enhancement, lower SNR% value, and more residual contrast at the end of the experiment are visualized in all brain subregions in the TBI-HT group, compared TBI-NT group, and in TBI-NT group, compared to both Sham-NT and Sham-HT groups. Similar TIC appearance also shows in all ipsilateral subregions **(A**, **B**, **C**, **E)** except cerebellum **(D)** in both TBI-HT and TBI-NT brain, compared to the contralateral subregions, but not in sham injury brains. HT-contra, contralateral subregion of TBI-HT rat brain; HT-IPSI, ipsilateral subregion of TBI-HT rat brain; NT-contra, contralateral subregion of TBI-NT rat brain; NT-IPSI, ipsilateral subregion of TBI-NT rat brain.

The contrast agent transport through the glymphatic pathway at a specific time point was illustrated in Figures 6, 7. As seen in Figures 6, 7, the contrast agent distribution started from the brain surface and then spread into deep brain tissue after cisterna magna infusion. When visually comparing the distribution pattern in four groups, TBI-HT rats showed the slowest diffusion among all animals. TBI-NT rats also exhibited decreased drainage patterns compared with sham-injured rats. Moreover, this drainage reduction in two TBI groups also showed an asymmetrical distribution in bilateral subregions, such as the cortex, hippocampus, and thalamus (Figures 6A–D). However, the asymmetrical distribution pattern was not found in the cerebellum and olfactory bulb (Figures 7A,B) and two sham groups. When comparing the TICs obtained from five brain subregions, it is evident that TBI-HT rats showed the lowest upslope, downslope, and plateau of TICs among the four groups (Figure 8). A similar TIC appearance was found in the TBI-NT group. The difference in TICs was also observed in all subregions of the ipsilateral injured brain compared to the corresponding contralateral brain subregions except cerebellum (Figure 8D), whether for the TBI-HT group or TBI-NT group.

**Figure 9:**
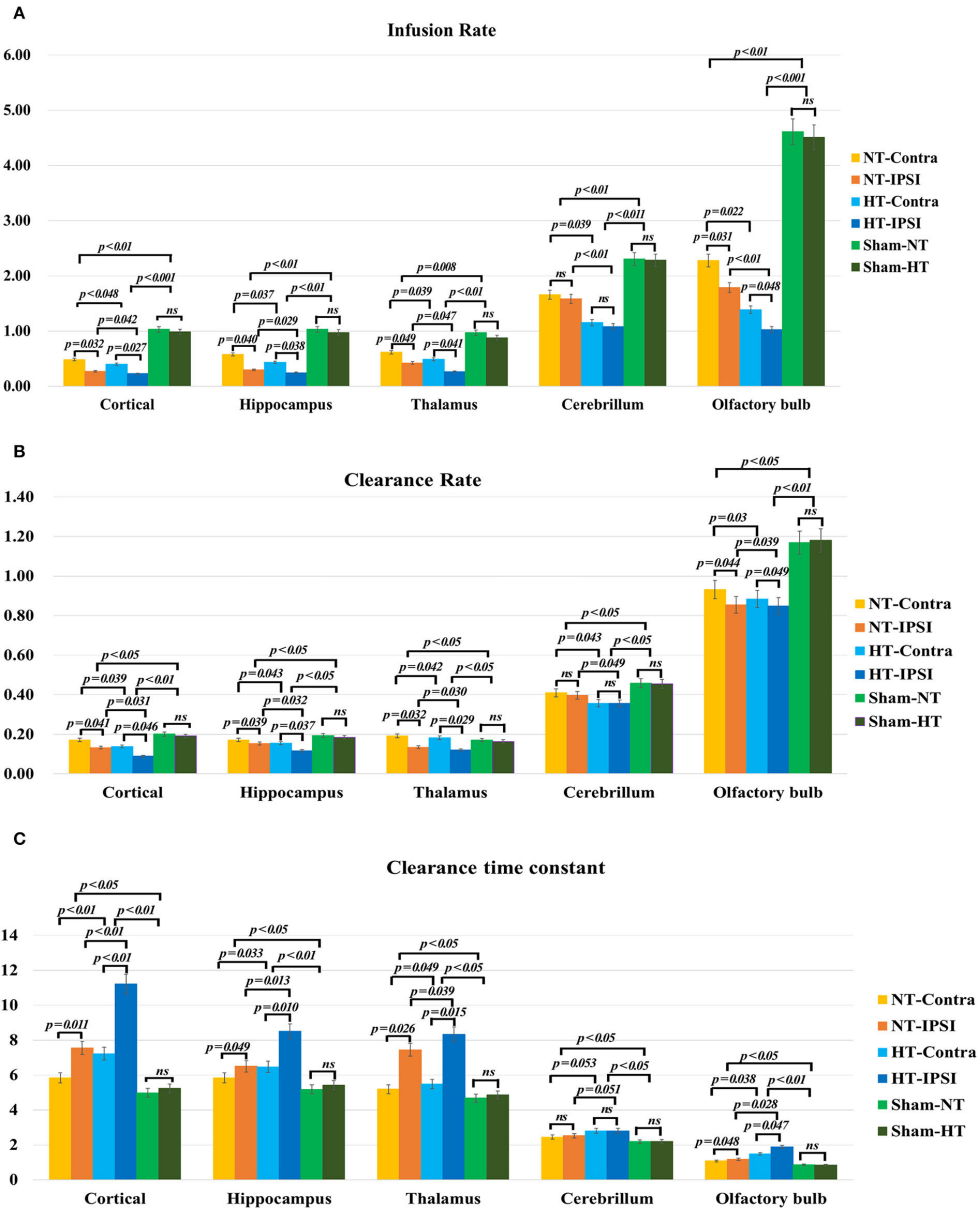
The quantitative analysis derived from TICs regarding the influence of TBI and hypothermia treatment on the glymphatic influx of low weight molecular contrast agent. Group comparison of infusion rate **(A)**, clearance rate **(B)**, and clearance time constant **(C)** in examined bilateral brain subregions, including ipsilateral and contralateral cortex, ipsilateral and contralateral hippocampus, ipsilateral and contralateral thalamus, ipsilateral and contralateral cerebellum, and ipsilateral and contralateral olfactory bulb. Within the major experimental period, a significant lower infusion and clearance rate and larger clearance time constant is detected in all brain subregions in the TBI-HT group, compared to the TBI-NT group, and in the TBI-NT group, compared to both Sham-NT and Sham-HT groups. Similar results are also found in all ipsilateral subregions except cerebellum in both TBI-HT and TBI-NT groups, compared to the contralateral subregions, but not in sham injury groups. Data in **(A–C)**: Sham-NT *n* = 6, sham-HT *n* = 6, TBI-NT *n* = 8, TBI-HT *n* = 8, pooled data from four independent experiments. All *n* values refer to the number of rats used, and the error bars depict the mean ± s.e.m. *P*-values were calculated by one-way ANOVA with Bonferroni's multiple comparison test, and P<0.05 indicates significance. HT-contra, contralateral subregion of TBI-HT rat brain; HT-IPSI, ipsilateral subregion of TBI-HT rat brain; NT-contra, contralateral subregion of TBI-NT rat brain; NT-IPSI, ipsilateral subregion of TBI-NT rat brain.

The quantitative kinetic parameters derived from TICs in Figure 8 are shown in Figure 9. The TBI-HT rats exhibited significantly decreased infusion rate, clearance rate value, and increased clearance time constant value in five representative brain subregions compared with TBI-NT and sham rats (Figures 9A–C, all *p* < 0.005). Similar significant differences were also found between TBI-NT and Sham rats and between the ipsilateral and contralateral brain subregions except for cerebellum in both TBI-HT and TBI-NT rats (Figures 9A–C, all *p* < 0.005). These differences were not found between the Sham-NT and Sham-HT rats. Moreover, the TICs-derived kinetics parameters also exhibited regional differences in animals. A larger infusion rate and clearance rate value and a shorter clearance time constant value was found in the cerebellum and olfactory bulb than in the cortex, hippocampus, and thalamus. Similar differences were also found between the cerebellum and olfactory bulb in both TBI-HT and TBI-NT rats, and in Sham-HT and Sham-NT rats.

### Perivascular AQP4 expression in TBI with hypothermia

**Figure 10:**
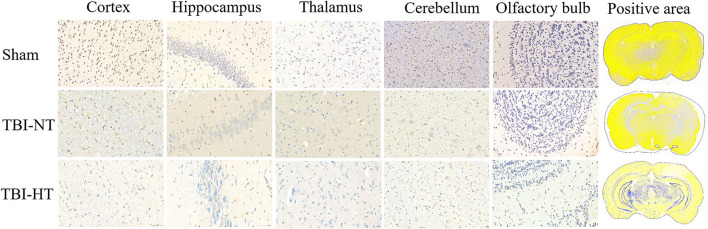
Representative AQP4 immunostaining photomicrographs and positive staining quantification for cortex, hippocampus, thalamus, cerebellum, and olfactory bulb from sham, TBI-NT, annnd TBI-HT. AQP4 expression across the brain of TBI-HT, characterized by color strength, is weaker than TBI-NT, and TBI-NT is weaker than sham control. The scale bar is 20 μm in the brain subregion and 1,000 μm in the whole brain.

**Figure 11:**
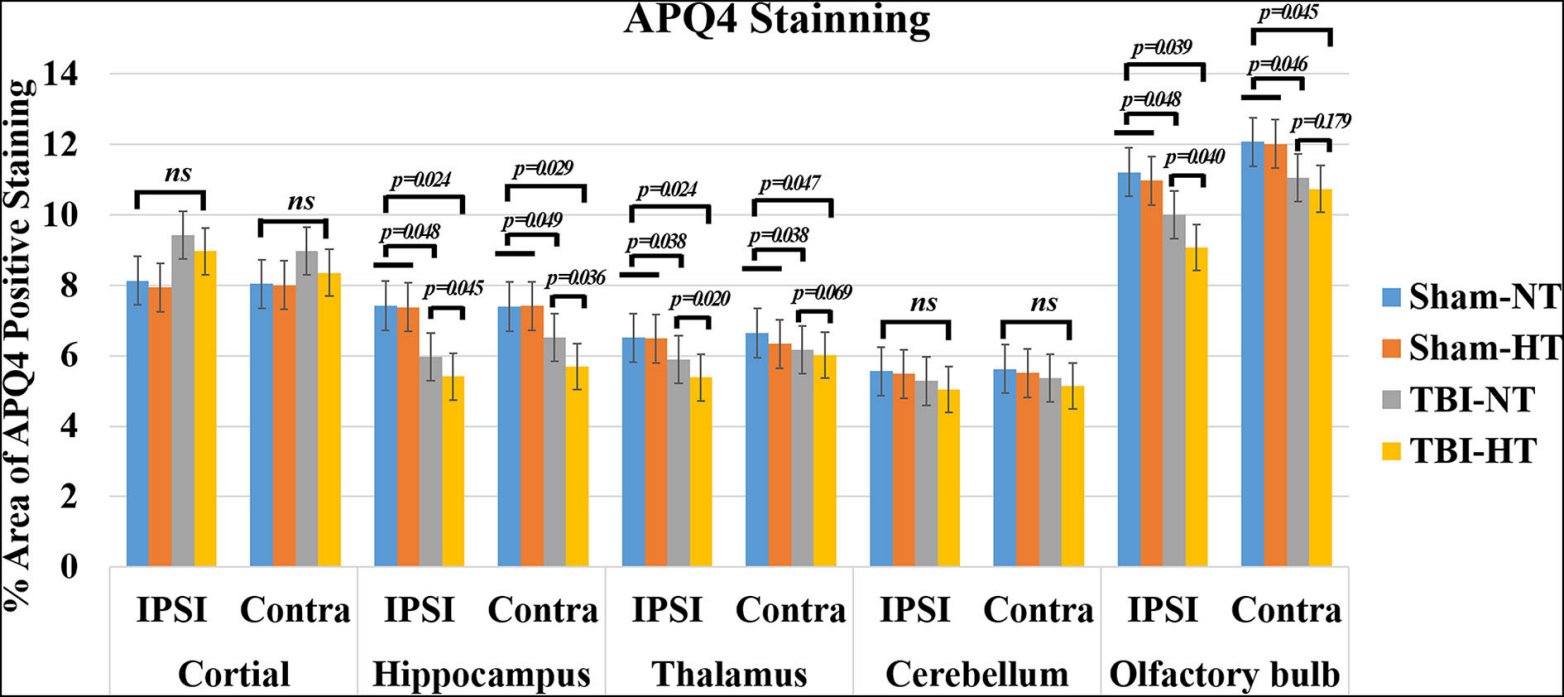
Quantification of bilateral, subregional APQ4 immunoactivity from sham, TBI-NT, and TBI-HT. The density of subregional AQP4 immunostaining is expressed as a percentage area of the subregion. Significantly decreased AQP4 expression, determined by mean positive area, was identified in all subregional brains in TBI-HT rats, compared with TBI-NT rats, and in TBI rats, compared with sham rats. Note: ns indicates no significance. Data in Figure 11: Sham-NT *n* = 6, sham-HT *n* = 6, TBI-NT *n* = 8, TBI-HT *n* = 8, pooled data from four independent experiments. All n values refer to the number of rats used, and the error bars depict the mean ± s.e.m. *P*-values were calculated by one-way ANOVA with Bonferroni's multiple comparison test, and *P* < 0.05 indicates significance. Contra, contralateral hemisphere; IPSI, ipsilateral hemisphere.

**Figure 12:**
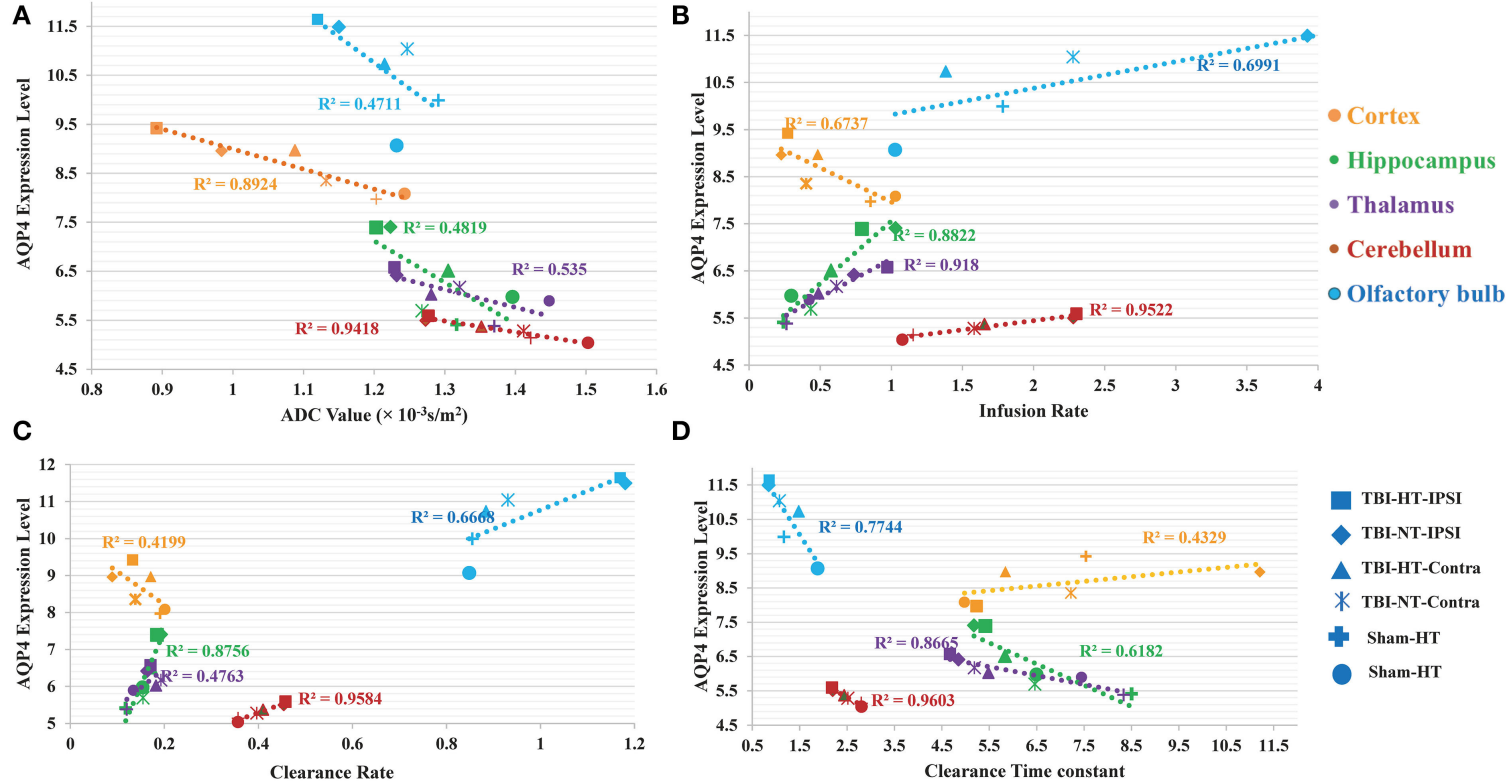
The correlation between APQ4 expression level with glymphatic dysfunction and ADC value is described in scatter plot graphs **(A–D)**. Scatter plot graphs show the APQ4 expression level in five brain subregions are linearly related to **(A)** ADC value **(B)** Infusion rate **(C)** Clearance rate, and **(D)** Clearance time constant of sham control, TBI-NT, and TBI-HT rats (*r*^2^ = 0.89, 0.48, 0.54, 0.94, 0.47 for ADC value, *r*^2^ = 0.67, 0.88, 0.92, 0.95, 0.70 for Infusion rate, *r*^2^ = 0.42, 0.88, 0.48, 0.96, 0.67 for Clearance rate and *r*^2^ = 0.43, 0.62, 0.87, 0.96, 0.77 for clearance time constant in cortex, hippocampus, thalamus, cerebellum, and olfactory bulb, respectively, all *p* < 0.01, Spearman's correlation test, *n* = 28).

Representative images depicting AQP_4_ expression are shown in Figure 10. Compared to TBI-NT, perivascular AQP_4_ expression in the TBI-HT, determined by the mean positive area, was significantly decreased in the ipsilateral or the contralateral hemisphere (Figure 11, Table 3, all *p* < 0.05). This AQP_4_ expression decrease was also found between the TBI-NT and sham animals. However, no difference was found between the sham-NT and sham-HT (Figure 11, Table 3, all *p* < 0.05). Compared to the perivascular AQP_4_ expression in the contralateral hemisphere, only TBI rats exhibited significantly reduced in the ipsilateral hemispheres except for the cerebellum, either for TBI-HT or TBI-NT. Moreover, perivascular AQP_4_ expression in TBI-HT and TBI-NT showed regional brain differences. The perivascularAQP_4_ expression in the cortex, hippocampus, thalamus, and cerebellum was significantly lower than in the olfactory bulb (Figure 11, Table 3, all *p* < 0.05). The correlation between APQ4 expression level with glymphatic dysfunction and ADC value is described in Figure 12. Scatter plot graphs show the APQ4 expression level in five brain subregions are linearly related to ADC value (*r*^2^ = 0.89, 0.48, 0.54, 0.94, 0.47), infusion rate (*r*^2^ = 0.67, 0.88, 0.92, 0.95, 0.70), clearance rate (*r*^2^ = 0.42, 0.88, 0.48, 0.96, 0.67), and clearance time constant (*r*^2^ = 0.43, 0.62, 0.87, 0.96, 0.77) of sham control, TBI-NT, and TBI-HT rats in cortex, hippocampus, thalamus, cerebellum, and olfactory bulb, respectively (all *p* < 0.01, Spearman's correlation test, *n* = 28).

**Table 3:** Quantitative analysis of AQP_4_ expression in TBI with hypothermia or normothermia.

**Brain regions/groups**	**Sham-NT**		**Sham-HT**		**TBI-NT**		**TBI-HT**	
	**IPSI**	**Contra**	**IPSI**	**Contra**	**IPSI**	**Contra**	**IPSI**	**Contra**
Cortex	8.13 ± 2.13	8.04 ± 2.25	7.94 ± 1.98	8.00 ± 2.41	9.42 ± 3.01	8.97 ± 2.87	8.96 ± 2.78	8.35 ± 2.73
Hippocampus	7.42 ± 2.46	7.39 ± 2.26	7.37 ± 2.14	7.41 ± 2.56	5.97 ± 1.46	6.51 ± 1.96	5.41 ± 1.45	5.69 ± 1.67
Thalamus	6.51 ± 1.56	6.64 ± 1.23	6.49 ± 1.65	6.34 ± 1.47	5.89 ± 1.23	6.17 ± 1.43	5.38 ± 1.20	6.02 ± 1.65
Cerebellum	5.56 ± 1.46	5.62 ± 1.43	5.48 ± 1.29	5.51 ± 1.36	5.28 ± 1.33	5.37 ± 1.25	5.04 ± 1.09	5.14 ± 1.21
Olfactory bulb	11.21 ± 3.27	12.07 ± 3.54	10.97 ± 2.98	12.01 ± 3.67	9.99 ± 2.45	11.04 ± 3.01	9.07 ± 2.56	10.73 ± 2.89
Total	38.83 ± 11.52	39.76 ± 12.41	38.25 ± 12.56	39.27 ± 13.01	36.55 ± 14.21	38.06 ± 13.89	33.86 ± 12.43	35.93 ± 12.47

Note: *P*-value represents statistical results from the analysis of the difference in p-tau and Beta-amyloid protein expression between the TBI-TH group and the control, the sham injury, and TBI-NT group using one-way ANOVA with Bonferroni's multiple comparison test.

## Discussion

Over the last decades, hypothermia has proven to significantly attenuate the free radical-induced increases in BBB permeability after injury, thus reducing brain edema (24, 25). Hypothermia could indeed attenuate brain edema, as demonstrated by smaller injured lesions and less vasogenic edema in most brain subregions in this study. However, in contrast to reducing cerebral edema, hypothermia exacerbated the reduction of efficiency of glymphatic transportation after TBI. This deterioration of glymphatic drainage was present brain-wide and showed hemispherical asymmetry and regional heterogeneity across the brain, associated with vasogenic edema. Moreover, our data show that glymphatic detention and vasogenic edema are closely associated with reducing perivascular AQP_4_ expression. The suppression of glymphatic transportation might eliminate the benefits of brain edema reduction induced by hypothermia and appears to provide an alternative pathophysiological factor indicating injury to the brain after TBI.

The TBI-induced cytotoxic and vasogenic edema can be demonstrated and quantified by non-invasive DWI. Our study showed that TBI-induced brain edema presented focally, globally, and heterogeneously. Compared to the corresponding area of cortex in sham animals, the controlled cortical impact area and adjacent cortex depicted hypointensity in the ADC map and decreased ADC value in both TBI-NT and TBI-HT (Figure 3, Table 1), revealing a focal and perifocal traumatic brain cytotoxic edema. However, in contrast to the injured cortex, other subregions, such as the bilateral hippocampus, thalamus, olfactory bulb, and cerebellum, delineate a significantly increased ADC value, revealing vasogenic edema. Consistently with previous studies, DWI could detect cytotoxic edema, which presented a decreased ADC and vasogenic edema and increased ADC (9, 26). Our study highlights that the vasogenic edema in most subregions, excluding the injured cortex, showed an asymmetrical and heterogeneous diffuse. The vasogenic edema in ipsilateral subregions, such as the hippocampus and thalamus, is significantly worse than that in corresponding contralateral subregions, demonstrated by a larger ADC value. However, similar differences in ADC value were not detected in the bilateral olfactory bulb and cerebellum. Furthermore, when treated with hypothermia, TBI-NT-induced brain edema could be eliminated, whether for cytotoxic or vasogenic edema, as demonstrated by decreased ADC volume of the injured lesion and decreased ADC value in subregions excluding the injured cortex in TBI-HT.

Our understanding of the pathophysiology of vasogenic brain edema is characterized by a protein-rich exudate from plasma extravasating from a disruption of the blood-brain barrier (9, 27). In contrast to these statements, we found that following intrathecal administration of Gd-DTPA, compared with sham animals, both TBI-HT and TBI-NT exhibited significantly reduced infusion rate, clearance rate, and increased clearance time constant in the parenchyma, including bilateral cortex, hippocampus, thalamus, and olfactory bulb. Our results confirm and extend the original model of glymphatic fluxes interruption following TBI (14, 19, 28–30). We also highlight that vasogenic brain edema is associated with the impairment of the glymphatic system after TBI. This finding agrees that CSF can rapidly enter the brain via the perivascular pathway following stroke and provide an essential source for the initial rise in brain water content (31). Compared with sham controls, TBI dramatically impaired influx of CSF tracer was present in the ipsilateral hemisphere and was observed in the contralateral hemisphere. Moreover, CSF tracer's infusion rate and clearance rate in the cortex, hippocampus, and thalamus are significantly lower than in the olfactory bulb and cerebellum, reflecting the regional heterogeneity in glymphatic transport function. These are associated with the corresponding regional measures in brain edema. Notably, compared with TBI-NT, our data demonstrate that TBI-HT leads to a significantly reduced infusion rate, clearance rate, and increased clearance time constant (Figures 8, 9) in the majority of examined regions, and these quantitative parameters are more closely related to the increased ADC value in TBI-HT than in TBI-NT in corresponding brain subregions. Thus, our results provide the first insight into the pathophysiological change of vasogenic brain edema following TBI with hypothermia, which is associated with impaired glymphatic fluxes in the brain. This impairment of glymphatic transportation exacerbated by hypothermia in the injured brain but not in the normal brain reminds us to assess hypothermia's application for TBI further. Thus, targeting glymphatic edema may offer a therapeutic strategy for treating acute brain pathologies.

The traumatic brain edema and associated glymphatic tracer detention induced by hypothermia might be related to a loss of perivascular AQP_4_ expression (Figure 12). In the present study, except for the lesion and its adjacent site, a significant reduction in AQP_4_ expression was observed across the brain in TBI-HT. This reduction was found in the ipsilateral and the contralateral hemisphere (Figure 11, Table 3). Moreover, a heterogeneous regional reduction of AQP_4_ expression was also found in the TBI-HT brain. Similar results were found in TBI-NT rats, in contrast to sham controls. We found that alterations in AQP_4_ expression in TBI-HT were associated with reducing contrast agent glymphatic transportation and vasogenic brain edema. This study highlights that brain injury could induce the immediate loss of AQP_4_ expression and worsen by hypothermia. Because aquaporin-4 facilitates CSF transport out of the perivascular space, a present study supported by studies that were lacking AQP_4_ exhibited a highly significant reduction in glymphatic clearance in the TBI brain (10, 32), suggesting that this pathway may remove fluid and other waste products such as b-amyloid protein from the central nervous system (10). Thus, normalizing AQP_4_ expression in the acute phase of TBI may provide a therapeutic avenue to eliminate edema and improve the clearance of interstitial wastes after TBI.

Hypothermia-induced slow drainage of contrast agent from the brain via glymphatic pathway could become an opposite factor to eliminating the brain edema from hypothermia for TBI. However, hypothermia could minimize brain edema and decrease elevated ICP, which might lead to perivascular space enlargement. However, recent studies showed that enlarged perivascular spaces might decrease the glymphatic system's influx and efflux function, causing impairment of the glymphatic system (33). Moreover, because CSF flux is driven by arterial pulsation (34, 35), hypothermia-induced systemic arterial hypertensive attenuation and cerebral blood flow reduction might lower the driven force to accelerate the circulation of CSF along the perivascular pathway, further impacting glymphatic function. Significantly, hypothermia might minimize the free radical generation and inflammatory processes. However, the impairment of glymphatic function could reduce the clearance of metabolic waste production after TBI and increase the accumulation of these metabolic substances, such as P-tau and aβ plaque, as well as small molecular wastes such as lactate, ions, water, and so on (36, 37). The retention of these metabolic wastes can adversely affect cell function and apoptosis, leading to secondary brain edema that can increase mortality and adverse clinical outcomes. The secondary brain edema induced by glymphatic retention was supported by the findings in subarachnoid hemorrhage (SAH) and high-altitude cerebral edema. Following SAH and hypoxic injury, blood components coagulation and cerebral vasodilation result in perivascular occlusion, which impairs CSF-ISF drainage (38, 39). Based on these findings, we deduced that the adverse effects induced by hypothermia could be improved by increasing glymphatic drainage. This assumption has been supported by a recently novel micro neurosurgical approach for managing moderate to severe TBI—the cisternostomy (40), via continuous CSF drainage from the basal cisterns to reverse the CSF shift edema and relieve the ICP to counteract the secondary brain injury. We propose that if the combination of hypothermia and cisternostomy were performed for TBI, the neurological outcome would be improved. A rigorous study should be designed to test this assumption in future.

There are several limitations to this study. First, we only performed a cross-sectional study on TBI with hypothermia at the acute phase; while multiple measurements at late time points following the injury need separate groups of animals, and longitudinal evaluations reveal the dynamic alterations of glymphatic function. Second, due to ADC being highly sensitive to detecting vasogenic and cytotoxic edema, intra-individual recording of highly resolved ADC and careful image mapping onto a standardized, high-resolution rat brain template would be necessary. Third, since the histopathologic examination occurred ~12 h post-injury, behavioral data and mortality rate collection post-injury were limited.

This study used dynamic CE-MRI with a low molecular weight contrast agent to characterize the glymphatic transport in TBI treated with hypothermia. We found hypothermia exacerbated the glymphatic detention following TBI in an asymmetrical and regional heterogeneity manner and related to vasogenic brain edema. These findings may indicate a negative effect of hypothermia in the restriction of brain edema after TBI. Importantly, this study is the first to examine the hypothermia impact on the glymphatic system in TBI, thus posing a novel emphasis on the potential role of hypothermia in TBI management.

## Data availability statement

The raw data supporting the conclusions of this article will be made available by the authors, without undue reservation.

## Ethics statement

The animal study was reviewed and approved by the animal care and experimental committee of the School of Medicine of Shanghai Jiao Tong University.

## Author contributions

QL, YL, and YB developed the study concept and design. MY, HM, FZ, XL, and CL collected imaging data and analyzed and interpreted the data. YB and MY wrote the first draft. QL, YB, and YL designed and revised this manuscript. All authors critically reviewed this manuscript, contributed to this article, and approved the submitted version.

## Funding

This work was supported by the National Natural Science Foundation of China (81900434), the Fudan University (IDF152064/016), and the National Natural Science Foundation of Shanghai (19ZR1408900) (Yingnan Bai); Innovative and Collaborative Project Funding of Shanghai University of Medicine & Health Science (SPCI-18-17-001), Joint Project of Shanghai Pudong New Area Health (PW2020D-14) (Mingyuan Yuan); National Natural Science Foundation of China (81871329), Shanghai Municipal Education Commission-Gaofeng Clinical Medicine Grant Support (2016427), Excellent discipline leader of Shanghai Municipal Planning Commission (2017BR041), and Shanghai key discipline of medical imaging (2017ZZ02005) (Yuehua Li).

## Conflict of interest

The authors declare that the research was conducted in the absence of any commercial or financial relationships that could be construed as a potential conflict of interest.

## Publisher's note

All claims expressed in this article are solely those of the authors and do not necessarily represent those of their affiliated organizations, or those of the publisher, the editors and the reviewers. Any product that may be evaluated in this article, or claim that may be made by its manufacturer, is not guaranteed or endorsed by the publisher.
